# Preliminary radiological result after establishment of hospital-based trauma registry in level-1 trauma hospital in developing country setting, prospective cohort study

**DOI:** 10.1016/j.amsu.2018.09.032

**Published:** 2018-09-27

**Authors:** Obada Hasan, Adeel Samad, Zohaib Nawaz, Tashfeen Ahmad, Zehra Abdul Muhammad, Shahryar Noordin

**Affiliations:** Department of Surgery, Section of Orthopedics, The Aga Khan University Hospital, Pakistan

**Keywords:** Trauma registry, Upper limb, Lower limb, Fracture, Radiology

## Abstract

**Introduction:**

Injuries are the second most common cause of disability, the fifth most common cause of healthy years of life lost per 1000 people and unfortunately 90% of mortality takes place in low-to middle-income countries. Trauma registries guide policymakers and health care providers in decision making in terms of resource allocation as well as enhancing trauma care outcomes. Furthermore data from these registries inform policy makers to decrease the rate of death and disability occurring as a result of injuries. We present our experience in setting up an orthopedic trauma registry and the first short term follow-up of radiological outcomes.

**Materials and methodology:**

Our study is a non-funded, non-commercial, prospective cohort study that was registered at Research Registry. The primary objectives of our study included assessing pattern of injuries in patients with upper and lower limb skeletal trauma presenting to our tertiary care academic university hospital and their respective outcomes. Data was collected by the musculoskeletal service line team members supervised by an experienced research associate and trauma consultants. The work has been reported in line with the STROCSS criteria.

**Results:**

A total of 177 patients were included in this analysis, of whom 101 (57.1%) patients had lower limb fractures, 64(36.1%) patients ad upper limb fractures and 12 (6.8%) patients had both upper and lower limbs involved. A total of 189 upper and lower limb fracture cases were recorded. 176 patients (93.1%) underwent surgeries and 13(6.9%) were managed nonoperatively. Roentgenographic outcomes were assessed using radiological criteria for each bone fractured.

**Conclusion:**

Establishing a trauma registry assists in identification of the pattern of injuries presenting to the hospital which helps in priority setting, care management and planning. This continuous audit of outcomes in turn, plays a significant role in quality improvement.

## Highlights

• At our side of the world, injuries are the 2nd most common cause of disability.
• They have more impact on developing countries, 90% of mortality takes place in low-to middle-income countries.
• Trauma registry guides policymakers and health care providers in the process of decision making to improve patient outcomes.
• It has an essential role in causing significant decline in rate of death and disability occurring as a result of injuries

## 1 Introduction

Presently, injury accounts for 10% of deaths and 15% of disability adjusted life years (DALYs), making it a major cause of morbidity and mortality globally [1]. According to a report from the World Health Organization (WHO) and World Bank, by 2020 injuries will account for 20% of all DALYs [2]. Injuries have a greater impact in low income countries and 90% of mortality takes place in low to middle-income countries (LMIC) [3]. A review of literature shows that injuries are the 2nd most common cause of disability, the 5th most common cause of healthy years of life lost per 1000 people and the 11th most common cause of premature death in Pakistan [1]. However the data on injury severity, outcome, and process of trauma care in Pakistan is sparse, which is a major hurdle in recognizing the gaps in trauma care [4].

Trauma registries are databases that use specific inclusion criteria to document trauma [5]. The data provided by these trauma registries guide policymakers in government and health care providers to rationalize resource allocation as well assessing multiple variables to improve patient outcomes. This plays a vital role in reducing harm and decreasing accidents, because the prevention policies work when the specific population is targeted at specific time and setting as informed by the data [6]. Moreover, successful implementation of trauma care systems which involves the use of trauma registries, has an essential role in substantially decreasing the rate of death and disability as a consequence of injuries [7]. A study conducted at our institution demonstrated that out of 18 trauma related deaths, 6 were preventable, 7 were potentially preventable, and 4 were non-preventable [8]. Furthermore, the clinical outcome data provided by trauma registries helps in establishment of protocols that ultimately improve the quality of care delivered to the trauma patients [7].

At our tertiary care academic hospital, an orthopedic trauma database of Upper and Lower Limb injury was established in 2015 to provide data regarding orthopedic injuries and their management. The aims of the study are to assess the pattern of injuries in upper and lower limbs and to evaluate their radiological and functional outcomes. Currently we present our experience in establishing this orthopedic trauma registry and the first short term follow-up of radiological outcomes in our patients.

## 2 Materials and methodology

This is a non-commercial study and it is registered at Research Registry with UIN 3466 and 3467 for lower limb fractures and upper limb fractures, respectively. The work has been reported in line with the STROCSS criteria [9].

The trauma registry was initiated after obtaining approval from Ethical Review Committee. Protocol was developed before study start-up and is available from corresponding author on request. Written informed consent was obtained from all patients as per Good Clinical Practice guidelines. In case of children or cognitively impaired participants, permission was obtained from parents or legally authorized representatives. The primary objectives of our study were to assess pattern of injuries in patients with upper and lower limb skeletal trauma presenting to our tertiary care academic university hospital and to evaluate their radiological outcomes.

Trauma injury patients were assessed by on duty orthopedic resident, admitted and operated upon by the surgical team consisting of orthopedic postgraduate trainee with minimum 3 years' experience and the trauma consultant. These patients were recruited from the hospital's emergency room as well as the in-patient and out-patient units of the hospital. All research processes were supervised by the trained research associate who has more than 4 years' research experience in patient recruitment and data management at orthopedic surgery department in close consultation with the trauma attendings. The following criteria were used for patient selection:

### 2.1 Inclusion criteria

1 All patients with upper limb (humerus, radius, ulna, hand bones including scaphoid, phalanges, metacarpal bones) fractures with or without additional trauma injuries
2 All patients with dislocations around shoulder, elbow, wrist, PIPJ and MPJ joints.
3 All patients with lower limb (pelvic, acetabulum, femur, tibia, fibula, ankle, metatarsal and phalanges) fractures with or without additional trauma injuries
4 Patients of all ages and genders.
5 Patients who signed written informed consent and were willing to participate in the study.

### 2.2 Exclusion criteria

1 Patients with pathological upper limb fractures secondary to tumour, metabolic bone disease, osteoporosis etc. without any trauma injury.
2 Patients with dislocations other than due to traumatic injury.

**Fig. 1:**
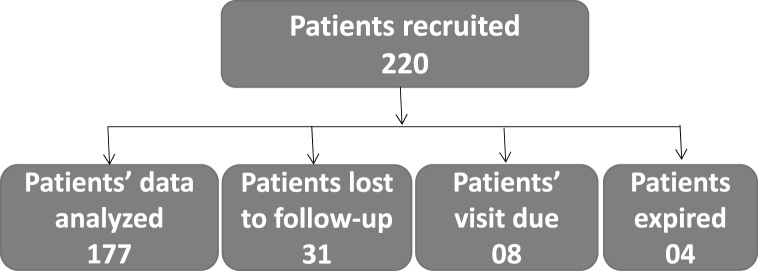
Patient's participation status.

Fig. 1 shows the patients recruited in study and those who were lost to follow-up.

The data was collected from patient's medical record by research associate using preset approved data collection form during hospital admission and at the follow-up visit outcomes were assessed at the clinic. Patients were followed at two weeks and scheduled for follow-up at six weeks, three-, six- and twelve-months after their initial visit. Patients were assessed by the operating surgeon who had minimum 5 years' experience as a consultant surgeon in our department. We assessed radiological outcomes for individual fractures using standardized scoring system [[10], [11], [12], [13], [14], [15], [16], [17], [18], [19], [20], [21]].

For this study we describe the radiological outcomes of the patients at 3rd follow up visit which was scheduled at 3 months ± 2 weeks following the surgery. We agree that radiological and clinical outcomes may differ. However, the clinical outcomes generally are inaccurate until full function is permitted by the treating surgeon, which depends on radiological union. Thus radiological union is a general prerequisite for good clinical/functional outcome. A longer follow-up is required for the latter, which is beyond the scope of the present paper. The forthcoming clinical and functional data will be reported as the long term follow-up in a separate report. We used SPSS version 22 to analyze the data.

## 3 Results

After excluding patients who were lost to follow-up, a total of 177 patients were included in this analysis (Fig. 1). Of these 101(57.1%) patients had lower limb fractures, 64(36.1%) patients had upper limb fractures and 12 (6.8%) patients had both upper and lower limbs involved. Thus a total of 113 lower limb and 76 upper limb fractures (189 upper and lower limb fractures) were managed. Out of the 76 upper limb fractures, 66 (86.8%) required surgery and 10 patients (13.2%) were managed nonoperatively. Among lower limb fractures, 110 (97.3%) patients were managed surgically and 3(2.7%) patients were managed non-operatively.

The sites of upper limb fractures included proximal humerus in 12 patients (15.8%), humerus shaft in 13 patients (17.1%), distal humerus in 13 patients (17.1%), radius ulna shaft in 19 patients (25%) and distal radius in 19 patients (25%). The age of patients ranged from 2 years to 83 years. The most common mechanism of injury was road traffic accidents (56.6%) followed by fall (36.8%), firearm injury (5.3%) and injury due to machinery (1.3%). The comorbids of the patients included Diabetes Mellitus (19.7%), hypertension (22.3%), dyslipidemia (1.3%), arthritis (1.3%) and ischemic heart disease (1.3%).The GCS at presentation was 13–15 in 73 patients (96.1), 9–12 in 1 patient (1.3%) and 3–8 in 2 patients (2.6%) (Table 1, Table 2).

**Table 1:** Clinical characteristics and demographics of patients with Upper limb fractures.

Fracture site with number of fractures		Proximal Humerus (12)	Humerus shaft (13)	Distal Humerus -including elbow (13)	Radius/Ulna Shaft (19)	Distal Radius (19)
**Injury type**	Fall	6	3	7	4	8
	Road traffic accident	5	10	5	12	11
	Firearm	1	0	1	2	0
	Machine	0	0	0	1	0
**Gender**	Male	8	9	11	14	13
	Female	4	4	2	5	6
**Comorbid**	DM	5	2	2	3	3
	HTN	6	2	2	4	3
	Dyslipidemia	0	0	0	0	1
	Arthritis	0	0	0	0	1
	Ischemic heart disease	1	1	0	0	0
**GCS**	9–12	0	0	0	0	1
	13–15	11	12	13	19	18

**Table 2:** Clinical characteristics and demographics of patients with lower limb fractures.

Fracture site with number of fractures		Proximal Femur (47)	Femur Shaft (13)	Distal Femur (11)	Tibia Plateau (16)	Tibia Shaft (26)
**Injury type**	Fall	36	4	0	1	0
	Road traffic accident	11	9	8	13	20
	Firearm	0	0	2	1	2
	Other	0	0	1	1	4
**Gender**	Male	24	10	8	15	26
	Female	23	3	3	1	0
**Comorbid**	DM	6	2	0	3	0
	HTN	12	1	1	1	0
	Ischemic heart disease	3	0	0	1	0
**GCS**	9–12	2	0	0	0	1
	13–15	45	13	11	16	25

Out of the 113 lower limb fractures, proximal femur fractures were 47 (41.6%), femur shaft 13 (11.5%), distal femur 11 (9.7%), tibial plateau 16 (14.2%) and tibia shaft fractures in 26 patients (23%).

Among 47 proximal femur fractures, 23 (48.9%) had intertrochanteric fractures (IT), 13 (27.7%) had neck of femur (NOF) fracture, and 2 patients (5.4%) had femur head fracture. Remaining patients had more than one of these proximal femur fractures. The majority of patients were males being 87 (74%) compared to 30 (26%) females. The age of these patients ranged from 14 to 74 years. Overall road traffic accident was the most common cause of injury accounting for 61 (54%) cases followed by falls in 41 (36.3%) of the cases. Hypertension was the most common comorbid in this group. At presentation, the GCS recorded showed that the majority lied in the range 13–15 in 110 out of 113 cases.

### 3.1 Radiological outcomes

We recorded 12 patients with proximal humerus fracture in the surgical group. Using Paavolainen's radiological criteria [15], outcomes were assessed in 11 patients. The humeral neck shaft angle was 130′ or greater in 7 (63.6%), 100–120′ in 2 (18.2%) and less than 100′ in 2 (18.2%) patients. The mean difference in humeral head height between 1st and 3rd visit was 4.37 ± 5. We recorded 1 patient in non-surgical group. The neck shaft angle was <100 on radiological assessment.

A total of 13 patients were recorded with humerus shaft fracture in the surgical group. Radiological outcomes were assessed in 5 patients (38.5%) using Stewart and Hundley's criteria [18,20]. The angulation was <10 in 2 and > 10 in 1patient whereas good alignment was noted in 2 patients. One patient was assessed in non-surgical group. Radiological assessment for angulation was done for 1 patient and showed good alignment.

A total of 13 patients were recorded with distal humerus fracture in the surgical group. Radiological criteria for distal humerus were assessed for children using Flynn's criteria [12,13]. For adults, only functional and clinical outcomes were measured.

We recorded 19 patients in the trauma registry with radius/ulna shaft fracture who underwent surgery. Radiological outcomes were assessed for union and loss of forearm rotation using Andersons Criteria [10]. We were able to assess for union in 10 and 12 patients with Radial and ulnar fracture respectively. All the 10 patients (100%) with radial fracture had excellent union at follow up visit of 6 weeks ( ±2 weeks). In the patients with ulnar fracture, 10 out of 12(83.3%) had excellent and 2 out of 12 had poor union. Sixteen radiographs revealed excellent rotation in 8 patients (50%), satisfactory rotation in 3(18.8%) patients and unsatisfactory rotation in 5 patients (31.3%).

We recorded 19 patients with distal fracture of radius in the surgical group using Stewart's assessment criteria [19]. Radiological outcomes were assessed in 8 patients and were excellent in 3, good in 4 and poor in 1 patient. We recorded 2 patients in non-surgical group. Only one patient was assessed for radiological outcome, which was good.

RUSH score [11] was applied to x-rays of hip fractures and outcomes was determined. On visit 3, 3 patients had a score of 30 and the rest had each scores of 3, 16, 21, 22 and 25. Table 3 shows systemic complications in proximal femur fractures treated surgically.

**Table 3:** In-Hospital Systemic complications for proximal femur fracture.

Complications	Frequency	Percent
None	34	72.3
Pulmonary	2	4.3
Renal/Urinary	3	6.4
Gastrointestinal	3	6.4
Neurological + pulmonary	1	2.1
Psychological	2	4.3
Gastrointestinal + pulmonary	1	2.1
Other-LFTs deranged	1	2.1
Total	47	100.0

Four out of 13 patients were assessed radiologically for femoral shaft fractures. We used Thorsten's criteria for this purpose [21]. Two had normal radiographic alignment whereas 1 had <5° and 1 had >10° angular deformity. One patient had marked shortening, 1 had shortening of <2 cm and 2 had no shortening after reunion. Three out of the 4 patients had a varus or valgus deformity of <5° whereas 1 had >10°.

Of the 11 patients with distal femur fracture we were able to assess only 2 patients with radiological outcomes. One patient had a deformity of <5° and loss of length of <1.5 cm whereas the other patient did not have any loss of length or deformity. Pritchett rating system and Scahtzker & Lambert Criteria was used to assess these patients [16].

We used Johner-Wruh's criteria [14] to evaluate tibial shaft fractures. Out of the 26 tibia shaft fractures, follow-up data is available for 14 patients. There was no deformity in 10 patients whereas 3 patients had a deformity ranging from 2 to 5° and only 1 patient had a deformity ranging 6–10°. One patient had a rotation of 6–10° with the rest 13 having a rotation of 5° or less. All 14 patients had shortening of less than 5 mm. Nine patients (64.3%) at the knee had normal angulation with 2 patients each having >75% and <75% at the knee. At the ankle, 7 had normal angulation whereas 2 had >75% and the remaining 5 had <75%. Similarly at the subtalar joint, 9 (64.3%) had >75% whereas 1 had >50% and 1 had <50%.

We were able to assess 7 patients out of the original 16 that presented with tibial plateau fractures using Rasmussen's scoring system [17]. Regarding depression, 4 had none while 2 had <6 mm and 1 had >10 mm. Six (85.7%) patients had no condylar widening with 1 patient having more than 10 mm. Similarly, 6 patients had no valgus or varus angulation with only 1 presenting with >20°.

## 4 Discussion

In low middle income countries, injuries account for five million deaths each year which is roughly equal to the combined number of deaths from HIV/AIDS, malaria and tuberculosis. Road traffic accidents constitute a major proportion of these injuries and cost more than US$ 500 billion annually. This is the reason why they have drawn a significant attention among all accidental injuries [22]. The recent advances in medical care have led to a higher survival rate of severely injured patients; however, this has left more individuals with long term disabilities [2].

A study conducted in seven largest tertiary-care hospitals in Pakistan revealed that each facility caters to 80–100 patients each day, which is a huge burden on Pakistan's underdeveloped health care system. The study reported that 68,390 patients presented with injuries from November 2010 to March 2011. The mechanism of injury was known in 19,102 patients of which 51.1% were road traffic injuries (RTIs) and 17.5% were falls [23]. Lack of road safety is a major contributing factor for the road traffic accidents and the literature reveals that the per capita yearly expenditure on road safety is just US$ 0.07 in Pakistan [24]. According to estimate by WHO the road traffic fatalities in 2013 were 25,781, with a 95% confidence interval of 20,979 to 30,582 [25]. Contrary to these reports, our numbers are low because this is a private university hospital with welfare support for non-affording patients. In this report we provide a short follow-up of our initial patients with upper and lower limb injuries.

Review of literature revealed a few studies done to assess the radiological outcome of upper limb fracture in trauma registries. Paavolainen reported the use of humerus shaft angle to determine the radiological outcomes. The humeral head-shaft angle was evaluated by radiographs the day after surgery, as well as after 12 and 24 months. The mean head-shaft angulation was 137.6° the day after surgery compared with 137.5° (p > 0.05) measured 12 months later, and 137.2° measured 24 months later (p > 0.05) [15,23]. Another retrospective study assessed the radiological outcomes of 87 patients with unstable displaced proximal humeral fractures treated with closed reduction and percutaneous pinning (CRPP) fixation. The fractures in all the patients were healed at an average time of 15.4 weeks (ranging from 12 to 43 weeks) [24]]. Rhodes and Aronson assessed radiological outcomes in patients with displaced distal radius fractures managed with 2.7 mm volar LCP fixation using Sarmiento's Modification of Lindstorm Criteria. Post-operative X-rays were analyzed immediately post-operatively, at six weeks and three months. The mean immediate post-operative radial shortening, decrease in radial deviation and loss of palmar tilt were 4.08 ± 2.23, 5.91 ± 4.01and 4.11 ± 3.29 respectively [26]. The corresponding values at last follow-up were 4.71 ± 2.31, 7.9 ± 5.13 and 4.91 ± 3.32 respectively. No statistically significant difference (p = 0.930) in radial shortening, decrease in palmar angulation and loss of radial deviation was seen till the final follow-up [27]. Another study done to assess the healing time radiologically revealed that the fractured wrists were evaluated using radiographs with two views (PA and lateral). We observed bone consolidation after a period ranging from 27 to 67 days, with a mean of 41 days [28].

Among adults below the age of 65, fractures to the lower extremity are responsible for over 200,000 hospital admissions each year [29]. Around 86% of patients admitted to a level 1 trauma center with multiple trauma have been reported to have fractures [30]. Although lower extremity fractures are rarely fatal, they often result in impairments (both temporary as well as permanent) that can affect the patient's general well-being and ability to function in a workplace [31]. Approximately half of the patients with a lower extremity fracture were reported to have partial disability 12 months after their injury [32]. In addition, 25% of patients with a lower limb fracture were unable to resume work after 1 year and one-fifth had not begun work 30 month after their injury [31].

Hip fractures are one of the most frequent scenarios seen in orthopedics service. Causes attributable include osteoporosis, falls, low body mass index, poly-pharmacy and cognitive impairment [33]. According to some estimates, they are expected to increase up to 21 million per year in 2050. Hip fracture prognosis is very poor with a one year mortality rate of up to 30% [34].

Due to large burden of trauma in Pakistan, we established an Upper and Lower Limb trauma care registry at our tertiary care academic university hospital in 2015 to assess the pattern of injuries. This study describes the radiological outcomes of the patients with upper and lower limb fractures at the 3rd follow up visit planned 3 months post operatively. The goal of the trauma registry is to follow the trauma patients over time. We also plan to assess the clinico-functional outcomes along with the radiological outcomes in the future. These results are of the initial fracture patients in the registry. Going forwards, we will also use this trauma registry to assess the current treatment practice along with the capacity and quality of trauma care at our hospitals to be analyzed and improved upon. Such patient outcome data will help us in reducing the morbidity and mortality through effective training of care providers and by improving performance of our hospital trauma care system.

## 5 Conclusion

Setting up a trauma registry helps identify the pattern of injuries presenting to the hospital. This data assists in priority setting, care management and planning. Orthopedic Section at our Hospital is substantially focused on trauma care as one of its major specialty areas. For trauma, the hospital infrastructure and processes contribute to the final outcome, in accordance with the actual clinical/surgical care provided to the patients. With this project, we intend to establish objective and reliable assessment of documented outcomes. This continuous audit of outcomes significantly enhances our quality improvement efforts.

### 5.1 Strengths

This ongoing prospective study and registry, to the best knowledge of the authors, is the first to be reported in the entire country. Data collation and analysis done by experienced group in the field supervised by attendings.

### 5.2 Limitations

The sample size of our study was relatively small to derive strong associations. We only reported the radiological outcomes at early follow-up since it is too soon to anticipate significant clinically relevant outcomes at this stage. Further research, including long term follow up with clinical and radiological outcomes for operated and conservatively treated fractures will help establish guidelines for resource scarce settings.

Provenance and peer review.

Not commissioned, peer reviewed.

## Ethical approval

Ethical Approval was obtained before start of study and it was given by out university Ethical Review Committee ERC of Aga Khan University Hospital.

Reference number: 3193-SUR-ERC-14.

## Sources of funding

NONE.

## Author contribution

**Obada Hasan:** Study design, Data management, writing the manuscript and final approval.

**Zohaib Nawaz:** Data collection, revising manuscript and final approval for publication.

**Adeel Samad:** Data and results analysis, writing the manuscript and final approval.

**Zehra Abdul:** Study design, protocol development and approval, data collection and analysis.

**Tashfeen Ahmad:** Study design, protocol development and approval, data management and final approval of manuscript.

**Shahryar Noordin:** Study design, result analysis, revising manuscript and final approval.

## Conflicts of interest

No conflict of interest.

## Trial registry number

UIN 3466 and 3467 for lower limb fractures and upper limb fractures, respectively.

## Guarantor

Obada Hasan, Tashfeen Ahmad, Shahryar Noordin.

## Appendix A Supplementary data

STROCSS checklist STROCSS checklist
